# Human movement modifications induced by different levels of transparency of an active upper limb exoskeleton

**DOI:** 10.3389/frobt.2024.1308958

**Published:** 2024-01-24

**Authors:** Dorian Verdel, Anais Farr, Thibault Devienne, Nicolas Vignais, Bastien Berret, Olivier Bruneau

**Affiliations:** ^1^ Complexité, Innovation, Activités Motrices et Sportives, Sport Sciences Department, Université Paris-Saclay, Orsay, France; ^2^ Complexité, Innovation, Activités Motrices et Sportives, Université d’Orléans, Orléans, France; ^3^ Laboratoire Universitaire de Recherche en Production Automatisée, Mechanical Engineering Department, ENS Paris-Saclay, Université Paris-Saclay, Gif-sur-Yvette, France; ^4^ Human Robotics Group, Department of Bioengineering, Imperial College of Science, Technology and Medicine, London, United-Kingdom; ^5^ ENS Rennes, Bruz, France; ^6^ Centrale Supelec, Université Paris-Saclay, Gif-sur-Yvette, France

**Keywords:** human-exoskeleton interaction, transparency, exoskeleton control, force control, performance metrics

## Abstract

Active upper limb exoskeletons are a potentially powerful tool for neuromotor rehabilitation. This potential depends on several basic control modes, one of them being *transparency*. In this control mode, the exoskeleton must follow the human movement without altering it, which theoretically implies null interaction efforts. Reaching high, albeit imperfect, levels of transparency requires both an adequate control method and an in-depth evaluation of the impacts of the exoskeleton on human movement. The present paper introduces such an evaluation for three different “transparent” controllers either based on an identification of the dynamics of the exoskeleton, or on force feedback control or on their combination. Therefore, these controllers are likely to induce clearly different levels of transparency by design. The conducted investigations could allow to better understand how humans adapt to transparent controllers, which are necessarily imperfect. A group of fourteen participants were subjected to these three controllers while performing reaching movements in a parasagittal plane. The subsequent analyses were conducted in terms of interaction efforts, kinematics, electromyographic signals and ergonomic feedback questionnaires. Results showed that, when subjected to less performing transparent controllers, participants strategies tended to induce relatively high interaction efforts, with higher muscle activity, which resulted in a small sensitivity of kinematic metrics. In other words, very different residual interaction efforts do not necessarily induce very different movement kinematics. Such a behavior could be explained by a natural human tendency to expend effort to preserve their preferred kinematics, which should be taken into account in future transparent controllers evaluation.

## 1 Introduction

Active upper-limb exoskeletons are promising devices mainly dedicated to robot-assisted rehabilitation (Pons, 2010; Jarrassé et al., 2014; Proietti et al., 2016; Mehrholz et al., 2020) and musculoskeletal disorders prevention (de Looze et al., 2016; Ajoudani et al., 2018; Nussbaum et al., 2019). The possibilities offered by active exoskeletons in general are also of interest for the investigation of human motor control theories (Selinger et al., 2015; Verdel et al., 2023a; b). Several elements have to be taken into account when dealing with human-exoskeleton interactions, like the mechanical design of the exoskeleton and the way the exoskeleton is controlled. One of the most basic behaviors that active exoskeletons need to provide to fulfill these applications’ requirements is *transparency*. This control mode consists in following the human movement without altering it (Pirondini et al., 2016; Just et al., 2018; Verdel et al., 2021b), which in an ideal case would result in null interaction efforts. Such a control mode can serve as a baseline to design other common controllers, typically weight support for rehabilitation applications (Just et al., 2017; 2020; Verdel et al., 2022a).

Transparency can be improved through several developments. First, the exoskeleton’s mechanical design, both in terms of the transmission (Garrec, 2010; Gopura et al., 2016; Hutter et al., 2018) and of the human-exoskeleton physical interfaces (Schiele and van der Helm, 2009; Jarrasse and Morel, 2012; Mallat et al., 2019; Verdel et al., 2022b; 2023c), is an important factor to minimize unwanted interaction efforts. The other necessary development to reach high levels of transparency is the control law definition. However, testing the degree of transparency of the design-control pair requires appropriate evaluation metrics that are sensitive to significant changes in the human behavior.

Several control laws have been proposed to compensate for a part of or all the exoskeleton’s dynamics, first using open-loop methods (Jarrasse et al., 2008b; Jarrassé et al., 2010; Jarrasse and Morel, 2012; Bastide et al., 2018). Although these methods led to a decrease in velocity (compared to movements performed without exoskeleton), they still conserved numerous properties of natural human movement (Bastide et al., 2018; Verdel et al., 2021b). More advanced methods introduced a force feedback control based on force/torque (FT) measurements at the level of human-exoskeleton interaction points (Kim et al., 2012; Just et al., 2018; Verdel et al., 2021a; 2022b; 2023c), which could also be coupled to hierarchical optimization (Zimmermann et al., 2019), inertial measurement units (Zimmermann et al., 2020) or learning based predictions (Jarrasse et al., 2008a). Nevertheless, few of these studies compared the performances of different controllers with an extensive method, as done in Just et al. (2018) between feedforward and disturbance observer controllers. Therefore, the effects of distinct levels of transparency on human movement remain unclear. In particular, the sensitivity of common human movement descriptors such as kinematics and electromyographic (EMG) signals with regard to different transparent controllers inducing different interaction efforts has not, to our knowledge, been investigated *per se*.

The evaluation of transparency is usually less investigated than the design of the controller structure. In particular, numerous studies involve few or no naive human participants (Kim et al., 2012; Zimmermann et al., 2019; 2020; Verdel et al., 2021b), which does not necessarily allow to draw definitive conclusions regarding the effectiveness of the controller and its impact on human movement. Furthermore, few studies have focused on developing extensive evaluation methodologies and comparing the relevance of performance metrics in the literature (Jarrassé et al., 2010; Pirondini et al., 2016). Finally, these studies did not include questionnaires to quantify the perceived ergonomics of the exoskeleton, which is necessary to ensure it is comfortable for potential users. The present study aims at comparing three different transparent controllers to movements performed outside the exoskeleton and between themselves to assess their effects on complementary performance metrics. In particular, the three controllers are evaluated in terms of interaction efforts, human movement kinematics and EMG signals during movements in a parasagittal plane. Furthermore, ergonomic feedback is collected from participants to ensure that the objective measurements are in line with their perception of the exoskeleton. Given a transparent exoskeleton should not alter natural human movements, the tested controllers will be compared to human performance outside the exoskeleton. Overall, we expect that our three controllers will induce significantly different interaction efforts, which could result in significantly different alterations of human movement. Therefore, lower interaction efforts should induce movement parameters significantly closer to those performed outside the exoskeleton than higher interaction efforts.

In the following, the employed methods are first described (Section 2). The design of the tested transparent controllers are first introduced (Section 2.1), followed by the evaluation task (Section 2.2) description. Then, materials and evaluation metrics are detailed (Section 2.3). Results of the evaluation of the tested transparent controllers are finally described (Section 3) and discussed (Section 4).

## 2 Materials and methods

### 2.1 Transparent controllers

As previously stated, the design of transparent controllers has already been investigated (Jarrasse et al., 2008a; Kim et al., 2012; Just et al., 2018; Zimmermann et al., 2019; 2020; Sommerhalder et al., 2020; Verdel et al., 2021a; b). These previous works suggest that there are two main steps to designing a functional transparent controller. First, an identification of the dynamics of the exoskeleton can be performed (Section 2.1.1). Second, force-torque (FT) sensors can be used to compensate for the identification errors and further minimize interaction efforts (Section 2.1.2). In the following, three transparent controllers based on either one or a combination of these two main steps, which should induce clearly different levels of transparency, are introduced.

#### 2.1.1 Open-loop compensation of dynamics

The compensation of the exoskeleton dynamics is the first step of the design of a transparent controller (Jarrassé et al., 2010; Jarrassé et al., 2012; Verdel et al., 2021b). Robot dynamics are generally formulated as in Eq. 1 (Gong et al., 2000; Siciliano et al., 2009; Moberg, 2010; Verdel et al., 2021b; 2022a),

$$M\left(q\right)\ddot{q}+C\left(q,\dot{q}\right)+G\left(q\right)+sign\left(\dot{q}\right)\mu_{C}+\mu_{v}\dot{q}=\tau_{m}$$ (1)

where **q**, $\dot{q}$ and $\ddot{q}$ are respectively the joints position, velocity and acceleration vectors, M is the inertia matrix, C is the vector of centrifugal and Coriolis terms, G is the gravity vector, ***μ***_***C***_ and ***μ***_***v***_ are respectively the Coulomb and viscous friction coefficients vectors and, finally, ***τ***_***m***_ is the motor torques vector. Usually, the different parameters, or more accurately their combinations into base parameters, can be identified with different exciting trajectories and nonlinear least square methods (Verdel et al., 2021b). In the present case, only the gravity effects were identified and compensated, thereby following a common procedure for this specific exoskeleton (Jarrassé et al., 2010; Jarrassé et al., 2012), which is due to the complex behavior of its transmission (Hamon et al., 2010; Verdel et al., 2021b). Furthermore, since this exoskeleton only includes optical encoders, the joints accelerations are not directly available. Consequently, the inertia cannot be fully compensated without knowledge regarding the trajectory. However, since the experiments were performed in a parasagittal plane at preferred velocity (see Section 2.2), gravity torques were predominant compared to the others efforts involved (i.e., friction and inertia). The open-loop compensation was thus achieved by generating a feedforward torque ${\hat{\tau }}_{m}=G\left(q\right)$ .

The identification procedure was composed of 20 static position maintenance, equally spread in the whole workspace of each involved joint. Joint positions and corresponding motor currents were used to identify the gravity torques using the “*lsqnonlin*” method of Matlab (MathWorks, Natick, MA, United States). The identification quality was manually verified by ensuring that the exoskeleton remained static in any position when unperturbed. This control mode, uniquely based on the open-loop compensation of the exoskeleton’s weight, will be referred to as OL in the rest of the present paper.

#### 2.1.2 Force feedback control

Although some recent exoskeletons include FT sensors (Just et al., 2018; Zimmermann et al., 2019; 2020), their introduction at the human-exoskeleton interaction points remains relatively rare. Such sensors are an asset when it comes to minimizing interaction forces. Nevertheless, they also increase the inertia and weight of the segments, which reduces the available torque and could have unwanted effects on human movement. Our version of the ABLE exoskeleton includes two FT sensors (one at the forearm cuff level and one at the arm cuff level). The implemented force feedback controller for our 2-degrees of freedom experiment in a parasagittal plane was defined as follows,

$$\tau_{FT}\left(t\right)=-K_{p}Lf_{e}\left(t\right)-K_{i}\int_{t_{0}}^{t}Lf_{e}\left(s\right)ds$$ (2)

where ***τ***_***FT***_ was a 2 × 1 vector representing the joint torques to apply at the shoulder and elbow levels respectively, $K_{p}$ and $K_{i}$ were 2 × 2 diagonal positive definite matrices representing the proportional and integral control gains respectively, and L was a 2 × 2 diagonal positive definite matrix containing the lever arms between the FT sensors (i.e., respectively the arm and forearm) and the proximal joint of the segment (i.e., respectively the shoulder and elbow). In the parasagittal plane, the latter matrix is constant because the elbow (respectively shoulder flexion/extension) joint of the exoskeleton is only controlled using the forearm (respectively the arm) FT sensor, which is therefore fixed in the joint frame. However, in the general 4-degrees of freedom case, it is dependent on the joint positions (**q**) of the exoskeleton because the FT sensors are not fixed in the joint frames. The initial (i.e., exoskeleton switch-on) and current times are represented by *t*_0_ and *t* respectively. Finally, $f_{e}={\left(F_{z,A},F_{z,FA}\right)}^{⊤}$ was the vector of normal forces applied on the human at the arm and forearm levels respectively, as measured by FT sensors (see Section 2.2 for the definition of the forces). These normal forces were the only ones generating admissible torques at the elbow and shoulder levels in the parasagittal plane. The controller objective was to track null normal forces to cancel them, which is why there is no desired force in Eq. 2. Finally, the proportional and integral gains were manually adjusted prior to the experiments for each joint so as to be maximized while avoiding instability, which optimizes the responsiveness of the system. This control mode, uniquely based on the closed-loop compensation of the normal interaction forces, will be referred to as CL in the rest of the present paper.

#### 2.1.3 Complete controller

**FIGURE 1:**
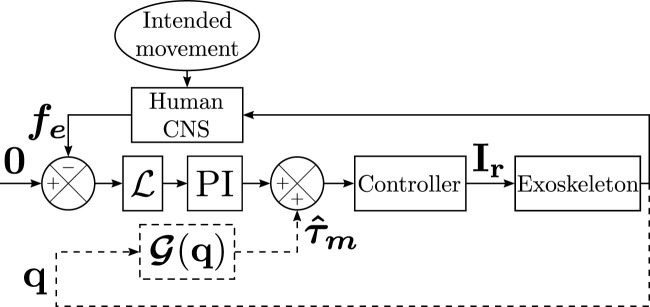
Structure of the controllers. The OL controller is represented with dashed lines and the CL controller by full lines. The OLCL controller is described by the complete structure. **I**_**r**_ is the vector of motor currents corresponding to the input torques.

The third transparent controller tested in the present paper is the combination of the OL and CL controllers, it will be referred to as OLCL. This controller should improve transparency compared to the two others by canceling identification errors through FT sensors and improving the tracking accuracy and reactivity by offsetting gravity efforts. Its structure is defined in Figure 1.

It is important to note that all the presented controllers generalize naturally to an arbitrary number of degrees of freedom.

### 2.2 Evaluation task

Four conditions were included and performed in a random order during the experimentation. Three conditions were performed inside the exoskeleton with the different transparent controllers (i.e., OL, CL and OLCL) and one without the exoskeleton (referred to as NE for No Exoskeleton), which served as a baseline for comparisons. As previously described, the human-exoskeleton physical interface at the forearm included a thermoformed orthosis, which locked the human wrist. Consequently, during the NE condition, participants wore a light splint preventing wrist movements to enable fair comparisons.

**FIGURE 2:**
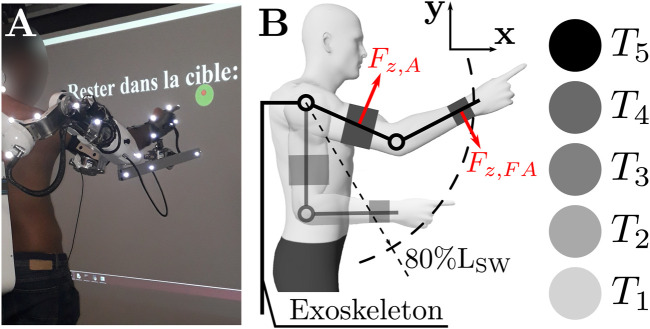
Description of the task. **(A)** Participant inside the target after the movement. The displayed message in French says: “Stay in target: *timer*”. The task is validated, therefore the target turned green. **(B)** Scheme describing the task and targets names. The controlled forces are represented and named in red. *L*_*SW*_ is the shoulder to wrist distance when the forearm is fully extended.

The task was composed of reaching movements in a parasagittal plane towards 5 targets projected on a screen (referred to as *T*_1_–*T*_5_, see Figure 2B). Given ABLE is a 4-degrees of freedom exoskeleton, its internal/external and abduction/adduction rotations of the shoulder were mechanically blocked. Before movements, participants held a reference position for 1.5 s, which was controlled in real time with the optoelectronic device. A message displayed on the screen informed the participant when the reference position was valid. The reference position was: the arm vertical, a 90° angle with the elbow and the index finger extended. Then a target, represented by a blue disk, appeared in front of the participant. A trial was considered valid whenever the participant had pointed at the target with the arm extended (i.e., the shoulder to wrist distance was at least 80% of the arm plus forearm length *L*_*SW*_). If the position was correct, the target turned green and the position was held for 1.5 s. A message displayed on the screen instructed the participants to remain in the target (see Figure 2A). Each target appeared 8 times, inducing a total of 40 reaching movements per condition. The target projection order was randomized. Movements were performed at the participant’s preferred velocity. At the end of each condition, a 2-min break was taken, during which the subject was asked to answer the previously introduced questionnaire.

#### 2.3 Materials and evaluation metrics

##### 2.3.1 Participants

A total of *N* = 14 participants (3 females) were involved in the experiment (mean age 26.33 ± 2.93 years old, mean height 1.76 ± 0.05 m, mean weight 72.87 ± 6.43 kg). Participants were healthy, right-handed adults without known neurological disorder or injury that could have impacted the experiment. Participants gave their written informed consent as required by the Helsinki declaration. The protocol was approved by the local ethical committee for research (CER-Paris-Saclay-2021-048).

##### 2.3.2 Recordings and evaluation metrics

###### 2.3.2.1 ABLE exoskeleton

The evaluation task was performed with the elbow and shoulder flexion/extension joints of an ABLE upper limb exoskeleton (Garrec et al., 2008). This exoskeleton includes 4 active joints (3 joints reproducing the human gleno-humeral joint and 1 reproducing the human elbow flexion/extension) actuated with a highly backdrivable system designed to maximize transparency (Garrec, 2010). Participants were connected to the exoskeleton at the arm and forearm levels by thermoformed orthoses. Two passive rotations and a passive translation were implemented at the level of the forearm to minimize unwanted interaction efforts and ensure a comfortable interaction (Jarrasse and Morel, 2012; Verdel et al., 2022b; 2023c). The posture of the participant is described in Figures 2A, B.

###### 2.3.2.2 Interaction efforts recording and analysis

Interaction efforts were measured by means of two 6-axes FT sensors (1010 Digital FT, ATI ^ⓒ^, maximum sample rate 7 kHz, used sample rate 1 kHz) placed at the connections between the human and the exoskeleton (i.e., at the arm and forearm, as previously specified). They were segmented based on the hand velocity computed using the robot joints velocity and common forward kinematics. The segmentation threshold was fixed at 5% of the peak velocity of the considered movement as it is a common practice in human motor control studies (Gentili et al., 2007; Berret et al., 2008; 2011; Gaveau et al., 2011; Verdel et al., 2021b; 2022a). All components of the human-exoskeleton interaction efforts were low-pass filtered (Butterworth, fourth order, cut-off frequency: 5 Hz).

The quality of the transparent controllers was assessed on the basis of their ability to minimize the amount of residual interaction efforts. First, a qualitative analysis of the average interaction efforts across participants and time was performed. Then, a quantitative analysis was conducted by computing the absolute maximum and absolute average of the controlled interaction efforts across participants. These parameters have different consequences with regard to the potential acceptability of the controllers. Indeed, the absolute average effort is known to be tied to the long-term acceptability whereas the absolute maximum effort is known to be tied to the short-term acceptability and pain at the connection level (Bessler et al., 2021; Verdel et al., 2023c).

###### 2.3.2.3 Kinematics recording and analysis

Human kinematics were recorded using an optoelectronic tracking system (10 Oqus 500 + cameras, 100 Hz; Qualisys, Gothenburg, Sweden). Six 10 mm reflective markers were placed on the participant’s right upper limb: head of acromion, medial and lateral epicondyles of the humerus, styloid process of the radius, head of the metacarpal, head of the proximal and distal phalanges of the index finger (see Figures 2A, B). Additional reflective markers were placed on the exoskeleton to help the real time labeling of the markers (see Figure 2A). The kinematics were assessed on the basis of the hand trajectory in the task space.

Recorded position data of each marker were low-pass filtered (Butterworth, fourth order, cut-off frequency: 5 Hz). Velocity and acceleration were obtained through numerical differentiation. Movements were segmented using a relative threshold fixed at 5% of the peak velocity of the considered movement as it is a common practice in human motor control studies (Gentili et al., 2007; Berret et al., 2008; 2011; Gaveau et al., 2011; Verdel et al., 2021b; 2022a).

The human movement kinematics were first qualitatively assessed by computing average trajectories across participants. Then, they were quantitatively assessed on the basis of movement duration (MD), curvature, peak velocity (PV, maximum absolute velocity during movement) and peak acceleration (PA, maximum absolute acceleration during the acceleration phase). The curvature, which is a common descriptor of spatial trajectories (Jarrassé et al., 2010), was defined as the maximum deviation of the trajectory with regard to a straight line in the task space, which was computed as in Eq. 3,

$$\text{Curv.}=\underset{t\in \left[t_{0},t_{f}\right]}{\max}\left(\frac{\left\|IT\times x\left(t\right)\right\|}{\left\|IT\right\|}\right)$$ (3)

where *t*_0_ (respectively *t*_*f*_) was the movement onset (respectively offset) time, **IT** was the vector supporting a straight path from the initial position of the hand in the task space (**x**_**0**_) to the target and **x**(*t*) represented the successive positions of the hand in the task space.

###### 2.3.2.4 Electromyographic recording and analysis

Six EMG sensors (Wave Plus, Wireless EMG, sample rate 2 kHz; Cometa, Bareggio, Italy) were placed on the right upper limb of participants. The following muscle activities were recorded: brachioradialis (flexors of the elbow), biceps brachii (flexor of the elbow and of the shoulder), triceps long (extensor of the elbow and of the shoulder) and lateral heads (extensor of the elbow), deltoid anterior (flexor of the shoulder) and deltoid posterior (extensor of the shoulder). The EMGs were placed according to the SENIAM recommendations (Hermens et al., 1999). Before placing the electrodes, the skin was locally shaved and cleaned with a hydro-alcoholic solution.

EMG signals of each muscle were first band-pass filtered (Butterworth, fourth order, cut-off frequencies: [20, 450] Hz), before being centered and rectified (Potvin and Brown, 2004). The human EMG activity levels in the four tested conditions were assessed by computing the root mean square (RMS) of these pre-processed EMG signals normalized by the maximum value reached during the experiment for each muscle separately. Then, the obtained values were averaged according to the functional muscle groups, following the categories previously defined: shoulder flexors and extensors and elbow flexors and extensors.

For visualisation purposes, the envelope of the signals was obtained by applying a low-pass filter (Butterworth, fifth order, cut-off frequency: 3 Hz) before normalizing by the maximum value reached during the experiment.

###### 2.3.2.5 Ergonomic feedback questionnaire

The subjective feeling of participants was evaluated using a Likert-scale of five items, ranging from 0 (i.e., strongly disagree with a negative statement and strongly agree with a positive statement) to 5 (i.e., strongly agree with a negative statement and strongly disagree with a positive statement) (Likert, 1932). Four statements were submitted to the participants:

• I felt fatigue in my right arm during the test.
• The task is difficult to achieve.
• It is comfortable to perform the task.
• It is difficult to reach the targets.

As a complement to this questionnaire, the physical effort felt by the participant was assessed using a CR-10 Borg-scale (Borg, 1998), ranging from 0 (i.e., resting) to 10 (i.e., maximal). In order to be comparable to other criterion, the grades reported by participants were rescaled between 0 and 5. Participants were informed of the anonymity of the questionnaire. Furthermore, they were asked to give an immediate answer and informed that there were no right or wrong choices, which is a common practice to limit the desirability bias (Patten, 2016).

###### 2.3.2.6 Statistical analyses

All statistical analyses were conducted with custom Python 3.8 scripts using the Pingouin package (Vallat, 2018). Since the data distribution was not normal [according to *Shapiro-Wilk* tests (Shapiro and Wilk, 1965)], nonparametric tests were conducted. First, Friedman tests were performed to assess possible main effects of the condition on the introduced human movement metrics. In the case of a significant main effect, Wilcoxon-Nemenyi *post hoc* comparisons were performed to assess which of the conditions induced significantly different behaviors. All the significance levels were set at *p* < 0.05. For all *post hoc* comparisons, the effect size was assessed by computing the Cohen’s *D*. Reported differences were considered as sufficiently pronounced in the case of effects of at least medium size (i.e., *D* > 0.4), otherwise the result would be flagged as weak.

## 3 Results

In this section, the results of the evaluation of the three tested controllers are described. First, their respective effects on the controlled interaction efforts (Section 3.1) is introduced. Then, the corresponding effects on human movement kinematics (Section 3.2) and EMG signals (Section 3.3) are investigated. Finally, the feedback of participants with regard to the perceived comfort is described (Section 3.4).

### 3.1 Interaction efforts

#### 3.1.1 Qualitative analyses

**FIGURE 3:**
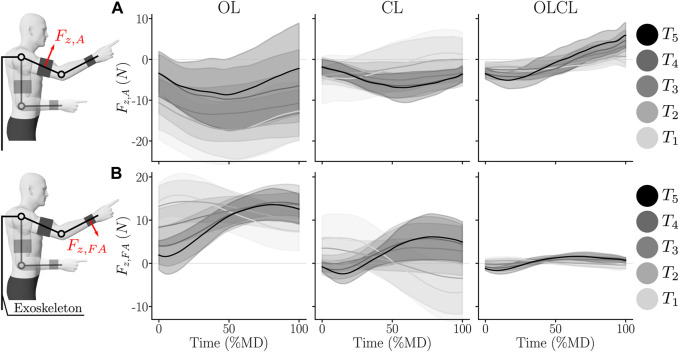
Average evolution of normal interaction efforts during forward movements for each condition (normalized in MD). Shaded areas represent the standard error across participants. **(A)** Normal interaction effort at the arm level. **(B)** Normal interaction effort at the forearm level.

The interaction efforts were first qualitatively assessed with an overview of their average temporal evolution for the different controllers and targets. The corresponding results are summarized in Figure 3 for each of the normal interaction force components.

The temporal evolution of the arm interaction force *F*_*z*,*A*_ suggests important differences between the conditions. In particular, *F*_*z*,*A*_ was mostly negative and of high magnitude in the OL condition and mostly negative and of smaller magnitude for the CL condition. For the OLCL condition, *F*_*z*,*A*_ was partly negative and then partly positive, with a lower magnitude than in the OL condition.

The average evolution of the forearm interaction force *F*_*z*,*FA*_ also suggests important differences between the conditions. In particular, the average evolution of *F*_*z*,*A*_ in the OL and CL conditions globally exhibited the same shape but was more centred around 0 N in the CL condition. In the OLCL condition, *F*_*z*,*A*_ was of a smaller magnitude than in the two other conditions and centred around 0 N.

#### 3.1.2 Quantitative analyses

**FIGURE 4:**
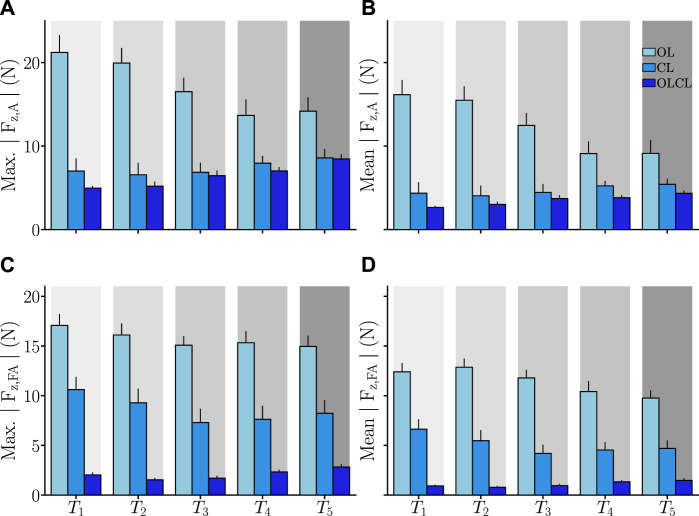
Average parameters computed on the controlled interaction efforts *F*_*z,A*_ and *F*_*z,FA*_. Error-bars represent the standard error across participants. **(A)** Maximum absolute interaction force at the arm level. **(B)** Average absolute interaction force at the arm level. **(C)** Maximum absolute interaction force at the forearm level. **(D)** Average absolute interaction force at the forearm level.

As previously mentioned, the effects of the controllers on interaction efforts were quantitatively assessed by computing the absolute maximum and average values of the normal force components. These results are summarized in Figure 4 for each target and each controller separately.

*Effects on* *F*_*z*,*A*_: Both the maximum and average absolute interaction efforts at the arm level seem to be impacted in the same manner by the different controllers. Friedman tests revealed significant main effects of the condition on both of these parameters (*W*_2_ = 0.65, *Q* = 18, *p* = 1.10^–4^ for the maximum and *W*_2_ = 0.71, *Q* = 19.9, *p* = 5.10^–5^ for the average absolute effort). In terms of maximum absolute *F*_*z*,*A*_, OL induced overall higher values than the other two controllers (in both cases: *p* < 4.10^–14^, *D* > 1.59). Despite CL inducing systematically higher average maximum absolute *F*_*z*,*A*_ than OLCL, no significant difference was found between these controllers on this parameter. In terms of average absolute *F*_*z*,*A*_, OL induced overall higher values than the other two controllers (in both cases: *p* < 7.10^–13^, *D* > 1.44). Although CL and OLCL were not significantly different on this parameter, it is worth mentioning that the *post hoc* comparison returned a relatively high effect size (*p* = 0.06, *D* = 0.45), thereby suggesting that the effect could become significant with a larger sample size. In sum, the OL condition induced higher interaction forces at the level of the arm than both the CL and OLCL conditions. Furthermore, despite some differences on average between CL and OLCL  no significant differences were found on its maximum and average values.

*Effects on* *F*_*z*,*FA*_: Both the maximum and average absolute interaction effort at the forearm level seem to be impacted in the same manner by the different controllers. Friedman tests revealed significant main effects of the condition on both these parameters (in both cases: *W*_2_ = 0.93, *Q* = 26.1, *p* = 2.10^–6^). In terms of maximum absolute *F*_*z*,*FA*_, OL induced significantly higher values than the other two controllers (in both cases: *p* < 2.10^–15^, *D* > 1.59). Furthermore, CL induced significantly higher maximum absolute interaction efforts than OLCL (*p* = 3.10^–23^, *D* = 1.84). In terms of average absolute *F*_*z*,*FA*_, OL induced significantly higher values than the other two controllers (in both cases: *p* < 2.10^–17^, *D* > 1.88). Furthermore, CL induced significantly higher maximum absolute interaction efforts than OLCL (*p* = 10^–23^, *D* = 1.65). In sum, OL induced the highest interaction forces at the level of the forearm, followed by CL and, finally, OLCL  which induced the lowest interaction efforts.

### 3.2 Kinematics

#### 3.2.1 Qualitative analyses

**FIGURE 5:**
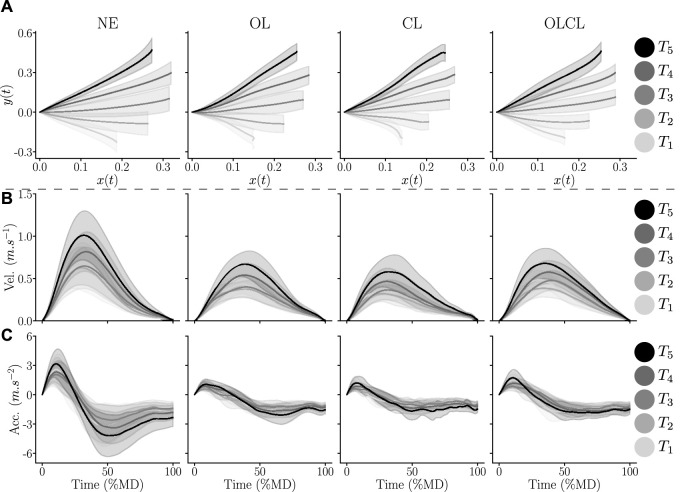
Average hand trajectories recorded for each of the targets across participants in each control mode. Shaded areas represent the standard error across participants. **(A)** Hand paths in the parasagital plane. **(B)** Hand velocity profiles, normalized by MD. **(C)** Hand acceleration profiles, normalized by MD.

The average trajectories recorded across the population for each of the 5 targets are represented in Figure 5.

The hand paths described in Figure 5A exhibit several clear differences between conditions. In particular, the average human path when reaching to *T*_1_ seems to be far more curved in the OL and CL conditions than in the NE and OLCL conditions. Overall, the path curvature seems less straight and smooth in the two former conditions than in the two latter ones, independently of the target reached.

Velocity (Figure 5B) and acceleration (Figure 5C) profiles seem to be strongly impacted by the exoskeleton independently of the control law and for all targets. In particular, the recorded velocities and accelerations reveal slower movements when wearing the exoskeleton. Furthermore, the acceleration profiles in the CL condition exhibit more variability than the other conditions during the deceleration phase. A finer-grained analysis is described below with the computation of the previously introduced kinematic parameters.

#### 3.2.2 Quantitative analyses

**FIGURE 6:**
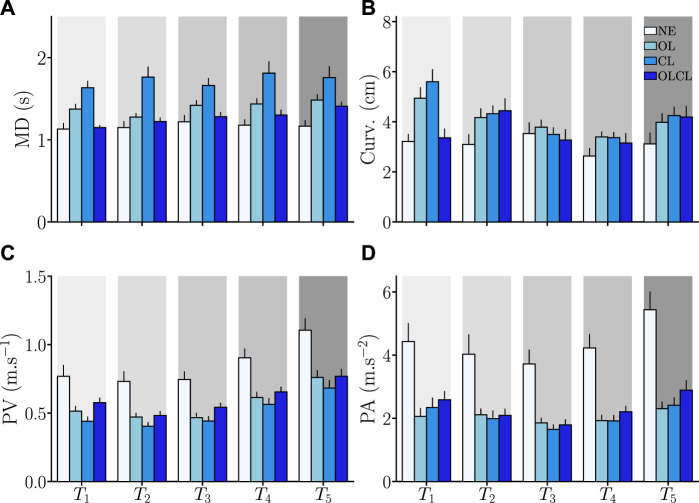
Average global and local kinematic parameters. Error-bars represent the standard error across participants. Parameters are computed separately for each target *T*_*i*_ and each condition. **(A)** Movement duration (MD). **(B)** Trajectory curvature. **(C)** Peak velocity (PV). **(D)** Peak acceleration (PA).

The obtained results regarding the kinematic performance metrics introduced in Section 2.3.2 are summarized in Figure 6, which presents the average values across participants.

*Movement duration* (MD): Overall, MD seems to be impacted by the exoskeleton, independently of the control mode. Nevertheless, this impact seems to be more important in OL and CL than in OLCL. A main effect of the condition was confirmed by a Friedman test (*W*_3_ = 0.77, *Q* = 32.1, *p* < 10^–6^). Wilcoxon-Nemenyi comparisons confirmed that participants were overall slower in CL than in all the other conditions (in all cases: *p* < 4.10^–7^, *D* > 0.92). Furthermore, participants were significantly slower in OL than in NE and OLCL (in both cases: *p* < 2.10^–3^, *D* > 0.57). Finally, participants were also slower in OLCL than in NE (*p* = 0.013, *D* = 0.42). In sum, the OLCL controller was shown to induce the smallest movement slowdown when compared to the OL and CL conditions.

*Curvature*: All the tested controllers seem to have an effect on movement curvature. The OL and CL conditions seem to induce either higher or comparable alterations with regard to NE than OLCL  depending on the aimed target. A Friedman test confirmed a main effect of the condition (*W*_3_ = 0.39, *Q* = 16.5, *p* = 9.10^–4^). Wilcoxon-Nemenyi comparisons confirmed that trajectories were overall more curved in OL and CL than NE (in both cases: *p* < 9.10^–5^, *D* > 0.66). A weak effect of the OLCL condition was also observed (*p* = 0.03, *D* = 0.37). In sum, OLCL was the best controller to conserve the natural human movements curvature.

*Peak velocity* (PV): The exoskeleton seemed to impact PV for all the tested controllers. This main effect was confirmed by a Friedman test (*W*_3_ = 0.74, *Q* = 31, *p* = 9.10^–7^). The CL condition induced significantly smaller PV when compared to NE and OLCL (in both cases: *p* < 6.10^–4^, *D* > 0.55). Furthermore, both OL (*p* = 4.10^–9^, *D* = 1.14) and OLCL (*p* = 3.10^–7^, *D* = 1) induced smaller PV when compared to NE. In sum, the exoskeleton clearly impacted PV and OLCL was slightly less impacting (reduction of: 28% ± 3%) than OL (reduction of: 34% ± 3%) and CL (reduction of: 41% ± 3%).

*Peak acceleration* (PA): The exoskeleton seemed to impact PA for all the tested controllers. This main effect was confirmed by a Friedman test (*W*_3_ = 0.68, *Q* = 28.4, *p* = 3.10^–6^). Overall, the three tested controllers induced smaller PA than NE (in all cases: *p* < 4.10^–11^, *D* > 1.2). Consequently, the exoskeleton clearly impacted PA and OLCL was slightly less impacting (reduction of: 47% ± 4%) on average than OL (reduction of: 53% ± 4%) and CL (reduction of: 53% ± 4%).

### 3.3 Impact on EMGs

#### 3.3.1 Qualitative analyses

**FIGURE 7:**
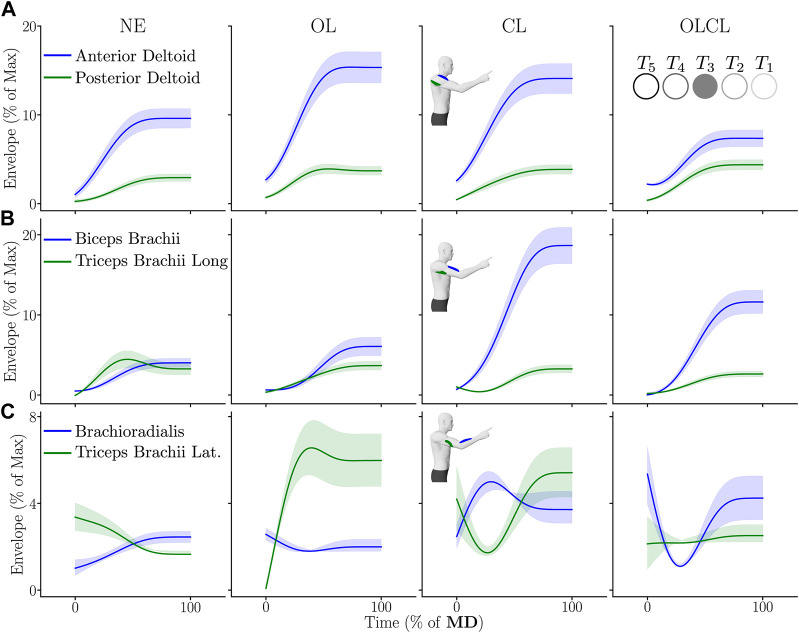
Average envelope of the activities of the different muscles (normalized by the maximum reached in the experiment) for a representative participant for *T*_3_. Shaded areas represent the standard error across participants. On each panel, a flexor muscle is represented in blue and an extensor muscle is represented in green. **(A)** Anterior and posterior deltoids. **(B)** Biceps brachii and triceps brachii long head. **(C)** Brachioradialis and triceps brachii lateral head.

The average envelopes of EMG signals recorded for a representative participant and for the target *T*_3_ are represented in Figure 7.

The envelopes described in Figure 7 exhibit clear variations of EMG amplitudes across conditions. In the OL condition, flexors activity was higher than in NE and OLCL for the anterior deltoid and than in NE for the biceps brachii. Furthermore, the lateral head of the triceps brachii was also more active than in the NE and OLCL conditions but comparable to the CL condition. In the CL condition, the envelope of flexors was overall higher than in all the other conditions. Finally, activities recorded in the OLCL condition were overall comparable to those recorded in the NE condition but with increases for the biceps brachii and the brachioradialis. In what follows, EMG activities are systematically investigated for all targets and participants through the RMS of the signals.

#### 3.3.2 Quantitative analyses

**FIGURE 8:**
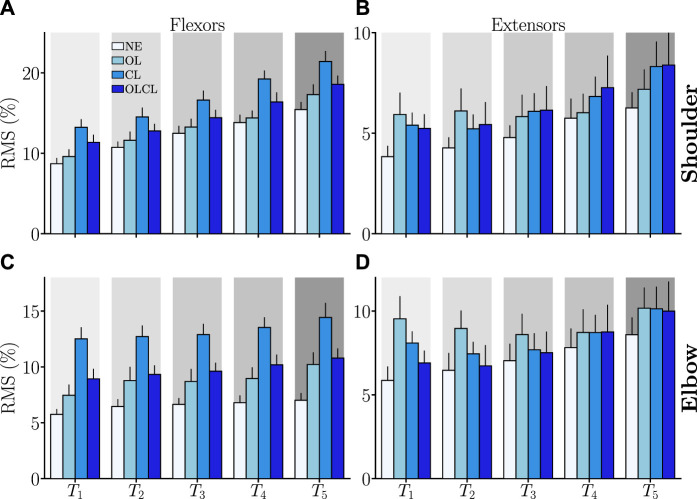
Average RMS of the activities of the different muscle groups (normalized by the maximum activity reached in the experiment) as a function of the aimed target. Error-bars represent the standard error across participants. **(A)** Shoulder flexors. **(B)** Shoulder extensors. **(C)** Elbow flexors. **(D)** Elbow extensors.

As previously introduced, the quantitative analyses conducted on the EMG signals were focused on the RMS, which reflects the average muscle effort expended by participants. The obtained results are summarized in Figure 8. Overall, EMG activity increased for all the transparent controllers under investigation despite the reduced movement velocity.

*Shoulder flexors*: All the tested controllers had an impact on the RMS of shoulder flexors. Consistently, a significant main effect of the condition was found on this parameter (*W*_3_ = 0.49, *Q* = 28.5, *p* = 10^–4^). Overall, CL induced higher levels of muscle activity than all the other conditions (in all cases: *p* < 10^–4^, *D* > 0.46). Furthermore, OLCL induced significantly more muscle activity than NE (*p* = 2.10^–3^, *D* = 0.58). Despite higher values with OLCL than with OL on average, these two controllers were not found to induce significantly different levels of shoulder flexors EMG activity. In sum, OL was the only tested controller that did not increase the level of muscle activity in shoulder flexors when compared to natural human movements and CL was the worst of the tested controller.

*Shoulder extensors*: Despite an increase for all targets and tested controller, no main effect of the condition was found on shoulder extensors RMS. This is probably due to the fact that all conditions performed when wearing the exoskeleton induced a relatively similar increase in this parameter when compared to NE.

*Elbow flexors*: All the tested controllers seemed to have an impact on the RMS of elbow flexors. Consistently, a significant main effect of the condition was found on this parameter (*W*_3_ = 0.68, *Q* = 28.4, *p* = 3.10^–6^). Overall, CL induced higher levels of muscle activity than all the other conditions (in all cases: *p* < 10^–6^, *D* > 0.92). Furthermore, both OL and OLCL induced significantly more muscle activity than NE (in both cases: *p* < 7.10^–4^, *D* > 0.7). Despite higher values with OLCL than with OL on average, these two controllers were not found to induce significantly different levels of elbow flexors EMG activity. In sum, all the tested controllers increased the elbow flexors EMG activity when compared to natural human movements, the CL controller inducing the greatest impact.

*Elbow extensors*: The tested controller seemed to have an impact on the RMS of elbow extensors. Consistently, a significant main effect of the condition was found on this parameter (*W*_3_ = 0.31, *Q* = 12.9, *p* = 0.005). Furthermore, OL induced higher levels of muscle activity when compared to NE (*p* = 0.003, *D* = 0.47). In sum, only OL induced a clear increase in the activity of elbow extensors when compared to natural human movements.

### 3.4 Ergonomic feedback

**FIGURE 9:**
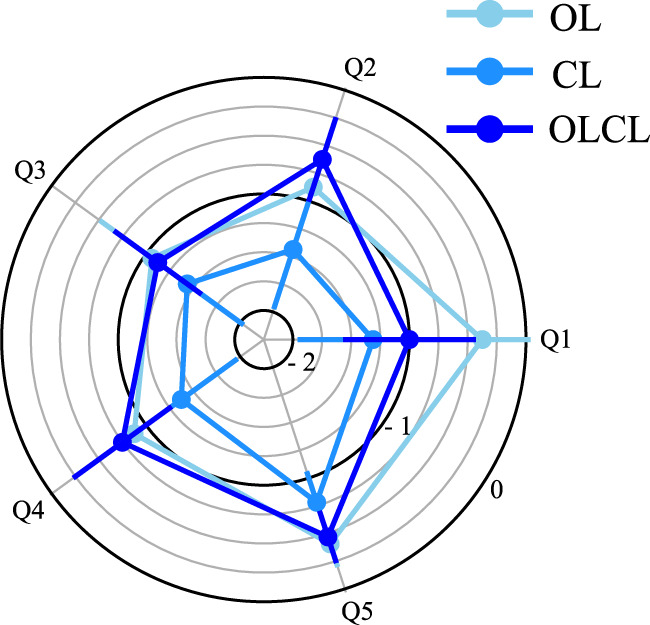
Average differences in responses to the questionnaire between NE and other conditions (i.e., OL, CL, OLCL). Error-bars represent the standard error across participants. **Q1**: Fatigue. **Q2**: Overall difficulty of the task. **Q3**: Comfort. **Q4**: Felt accuracy constraint. **Q5**: Physical effort.

The results of the performed investigations regarding the ergonomic feedback of participants introduced in Section 2.3.2 are summarized in Figure 9.

Overall, all conditions involving the exoskeleton seem to increase the grades reported by the participants on all the tested criteria, which correspond to a decrease in comfort and an increase in the felt difficulty. Main effects and comparisons were analyzed separately for each statement submitted to the participants.

*Felt fatigue:* A main effect of the condition was found on the reported fatigue (*W*_3_ = 0.36, *Q* = 17, 12, *p* = 7.10^–4^). Further comparisons showed that CL and OLCL implied a higher level of reported fatigue than NE (in both cases: *p* < 0.011, *D* > 0.96). Reported fatigue was not significantly affected by OL when compared to NE. Furthermore, CL performed significantly worse than OL (*p* = 0.016, *D* = 1). In sum, OL was the less impacting in terms of reported fatigue, followed by OLCL and finally CL.

*Difficulty of the task:* A main effect of the condition was found on the reported overall difficulty of the task (*W*_3_ = 0.46, *Q* = 22.23, *p* = 6.10^–5^). Further comparisons showed that all conditions involving the exoskeleton made the task more difficult when compared to outside the exoskeleton (in all cases: *p* < 0.01, *D* > 1.03). Furthermore, CL was shown to perform significantly worse than OLCL on this criterion (*p* = 0.014, *D* = 0.99). In sum, all the tested conditions impacted the felt difficulty, CL being the most impacting and OLCL being the less impacting on average.

*Comfort of the task:* A main effect of the condition was found on the reported comfort in the task realization (*W*_3_ = 0.44, *Q* = 21.23, *p* = 9.10^–5^). Further comparisons showed that the exoskeleton tended to decrease comfort independently of the condition. Indeed, all the tested conditions with the exoskeleton were significantly different of NE (in all cases: *p* < 8.10^–4^, *D* > 1.37), but no significant difference was found between them.

*Accuracy constraint:* A main effect of the condition was found on the felt accuracy constraint (*W*_3_ = 0.44, *Q* = 21.36, *p* = 9.10^–5^). Further comparisons showed that all conditions involving the exoskeleton increased the felt accuracy constraint when compared to outside the exoskeleton (in all cases: *p* < 0.008, *D* > 1.04). No significant differences were found between OL, CL and OLCL. Nevertheless, it is worth mentioning that, on average, CL increased the reported accuracy constraint when compared to OL and OLCL.

*Physical effort:* A main effect of the condition was found on the level of physical effort induced by the task (*W*_3_ = 0.59, *Q* = 28.38, *p* = 3.10^–6^). Further comparisons showed that all conditions involving the exoskeleton induced higher levels of effort when compared to outside the exoskeleton (in all cases: *p* < 0.003, *D* > 1.24). The CL condition was also shown to perform worse than OL for this criterion (*p* = 0.024, *D* = 0.91). In sum, CL induced the highest level of physical effort required to perform the task and OL and OLCL also increased it when compared to outside the exoskeleton.

## 4 Discussion

The present paper evaluated different transparent controllers and their respective effects on human movement. Transparency is one of the most basic and important features that should be provided by exoskeletons. This control mode is both a necessity in preventing musculoskeletal disorders at work, for instance during load-carrying tasks (Baser and Konukseven, 2012), and in exoskeleton-assisted rehabilitation protocols (Pirondini et al., 2016; Proietti et al., 2016; Zimmermann et al., 2020). The effects of distinct levels of transparency on human movements have not, to our knowledge, been extensively investigated in the literature. Such investigations could provide a deeper understanding of how human participants deal with an imperfect transparency of the exoskeleton, which is unavoidable in practice. In particular, it could shed light on the human movement parameters that are most impacted by the exoskeleton. In the following, we discuss the obtained results in terms of interaction efforts, kinematics, EMG signals and ergonomic feedback with our three transparent controllers and outside the exoskeleton (NE).

The three tested transparent controllers (i.e., OL, CL and OLCL) were first evaluated on the basis of the residual interaction forces occurring at the human-exoskeleton interfaces. In particular, it was shown that the OL controller induced higher interaction forces at both the arm and forearm interfaces than the CL and OLCL controllers. Quantitatively, both the maximum and average absolute interaction forces at the arm level were between two times and three times higher in OL than in CL and OLCL  with peaks above 20 N. It was also shown that, despite some differences in their temporal evolution, arm interaction forces were comparable in terms of magnitude for the CL and OLCL controllers. This could denote a predominant effect of the arm segment inertia, which was not fully compensated, on interaction efforts at this level. Finally, the interaction efforts measured at the forearm level were clearly lower for the OLCL controller than for the other two. Quantitatively, both the maximum and average absolute interaction forces at the forearm level were around ten times lower in OLCL than in OL and five times lower in OLCL than in CL, with peaks around 2 N and average values around 1 N. Therefore, in terms of interaction efforts, the OLCL controller is by far the most transparent of the three tested controllers. Nevertheless, these results do not allow to capture all human movement alterations due to each controller. Indeed, a controller could induce drastic changes of the kinematic strategy of the user even though it is associated with small interaction efforts.

In particular, a kinematic analysis is often a necessary step to clarify these alterations (Jarrassé et al., 2010; Pirondini et al., 2016; Bastide et al., 2018; Verdel et al., 2021b). Such an analysis first showed that movements performed with controllers including only one of the control components (i.e., open-loop compensation of gravity OL or interaction force minimization CL) induced an overall higher movement duration (MD) and trajectory curvature than the complete controller OLCL. Then, the controllers were evaluated in terms of their impact on peak velocity and peak acceleration. Interestingly, although the OLCL controller tended to limit the overall movement slowdown when compared to the other two controllers, the magnitude of the improvement is not as obvious as expected given the important differences reported in terms of interaction efforts. This could be explained by recent advances in the understanding of human movement invigoration via the minimum time-effort theory. Indeed, recent works showed that participants can be prone to spend significant amounts of energy, and withstand high interaction efforts, in order to save time (Verdel et al., 2023b). Here, participants reduced their speed likely in relation with the associated effort, as predicted by a time-effort trade-off (Bastide et al., 2018). Furthermore, they also preserved relatively straight hand paths towards the targets but performed at lower velocity, which suggests that humans try to preserve their overall kinematics as already well-known in reaching movements perturbed by velocity dependent force fields (Burdet et al., 2001; Franklin et al., 2003; Burdet et al., 2006). Moving more quickly could increase interaction efforts and energy expenditure, such that participants may have found a compromise in this task. Such a natural tendency could induce safety risks in terms of the skin condition and the development of musculoskeletal disorders during a prolonged usage of the exoskeleton (Bessler et al., 2021). Furthermore, it should be noted that the relatively noisy acceleration and velocity profiles observed in CL could be detrimental to accuracy, which is known to modify movement trajectories and to decrease velocity (Fitts, 1954). Such constraints are mainly due to the inherent delay induced by the integral term in the force-feedback control. Indeed, this term tends to delay the consideration of the participant’s acceleration variations by the exoskeleton, in particular in the absence of an open-loop compensation of the exoskeleton’s weight. Finally, these analyses tend to show that kinematic parameters alone are not sufficient to assess the differences between several transparent controllers. Such a result is important because numerous exoskeletons do not embed FT sensors to evaluate the magnitude of interaction forces (Sugar et al., 2007; Puchinger et al., 2018; Samper-Escudero et al., 2022), mainly for economic or compactness reasons.

The analyses conducted on both kinematics and interaction efforts suggest a better transparency of the OLCL controller. Nevertheless, it is important to assess the level of required muscle activity induced by the transparent controllers. In the present paper, the activity levels were assessed by means of the RMS of the EMG signals. In the introduced task, the agonist muscles are the shoulder flexors and the elbow extensors. Overall, CL induced higher levels of shoulder agonists activity than OL and OLCL, while also inducing the slowest movements. Furthermore, despite a trend of higher RMS on average with OLCL than with OL, these two controllers did not induce significantly different levels of shoulder agonists activity. Finally, OL induced higher levels of elbow agonists activity than CL and OLCL. The activity of elbow antagonists (i.e., biceps brachii and brachioradialis) induced by CL was higher than with OL and OLCL, while OL and OLCL were comparable. Therefore, it can be concluded that CL induced higher levels of muscle activity and for longer periods than OLCL, while OL induced comparable or higher levels of muscle activity for slightly longer periods than OLCL. Furthermore, the high activity levels of both agonists and antagonists in CL is consistent with accuracy constraints, which are known to induce muscle co-activation (Gribble et al., 2003). Finally, activity levels were overall higher when wearing the exoskeleton than in NE. These results are consistent with those previously obtained, in particular with regard to elbow flexors activity Pirondini et al. (2016). However, previous experiments also reported modifications in muscle synergies, which cannot be properly assessed with our limited number of measured muscles. Our observations, combined with the relatively small differences observed on kinematics between the different controllers, are consistent with a compensation of higher interaction efforts at the price of higher muscle activity. It must be noted that if a heavy load was carried in the task, we would probably have found that EMG activity in NE is higher than in any transparent condition. In our task, it is important to remind that no load was carried by the exoskeleton or the human, and that we simply analyzed nominal arm movements.

Overall, these results show the necessity of combining both an open-loop compensation of the exoskeleton dynamics and force feedback control to improve transparency. Nevertheless, these objective metrics do not allow to conclude about the preferences of the participants, which is why subjective feelings are an important information to collect.

Interestingly, the important differences reported on objective measurements do not seem to induce very different ratings by the participants. Indeed, although the CL controller performed significantly worse in all the tested questionnaire parameters, the differences in ratings are not high. Furthermore, the OL condition was rated equivalently to the OLCL condition, whereas it induced the highest interaction efforts between the tested controllers. In particular, although it was not significant, participants found the OLCL controller more fatiguing than the OL controller on average. This is probably due to the limited identification conducted which induced a stronger resistance to movement onset in the OL control mode, thereby compensating for a part of the human gravitational torque during static position maintenance. This compensation, unwanted in transparent mode, might have been used by participants to limit their energetic expense, in particular while pointing above their shoulder as for *T*_5_. Indeed, such postures are known to be non ergonomic (McAtamney and Corlett, 1993). It should be noted that these trends could change during prolonged usage, which emphasizes the importance of long-term field studies in the future.

Given all the presented results, future works should focus on accurately compensating the inertial torques induced by the exoskeleton. Such compensations need to be performed with the help of predictive techniques to be efficient (Lamy et al., 2009), which falls in the field of human intention detection and prediction. Such predictions could be based on computational motor control models (Berret et al., 2011), learning techniques (Jarrasse et al., 2008a), movement primitives (Jamsek et al., 2021; Gomes et al., 2022) or EMG data (Trigili et al., 2019; Treussart et al., 2020) for example,. The present work highlights the performances that can be expected for several transparent controllers, which can guide a choice of controller depending on the application and availablematerials. Furthermore, the extensive evaluation performed allowed a deeper understanding of the alterations of human movement produced by the exoskeleton. In particular, it was shown that human arm’s kinematics and EMG signals alone cannot properly discriminate between different levels of transparency, which is due to the human tendency to compensate for the controller imperfections. Interestingly, the employed evaluation method could be useful to evaluate other important control modes of exoskeletons, such as weight compensation (Just et al., 2017; 2020; Verdel et al., 2022a), which has been recently shown to impact the introduced parameters in the field of human motor control (Verdel et al., 2023a).

## Data Availability

The original contributions presented in the study are included in the article/Supplementary material, further inquiries can be directed to the corresponding author.
